# Risk of exacerbation following pneumonia in adults with heart failure or chronic obstructive pulmonary disease

**DOI:** 10.1371/journal.pone.0184877

**Published:** 2017-10-13

**Authors:** Rebecca Bornheimer, Kimberly M. Shea, Reiko Sato, Derek Weycker, Stephen I. Pelton

**Affiliations:** 1 Policy Analysis Inc. (PAI), Brookline, MA, United States of America; 2 Boston University Schools of Medicine and Public Health, Boston, MA, United States of America; 3 Pfizer Inc., Collegeville, PA, United States of America; 4 Boston Medical Center, Boston, MA, United States of America; National and Kapodistrian University of Athens, SWITZERLAND

## Abstract

**Background:**

Recent evidence demonstrates increased short-term risk of cardiac complications and respiratory failure among patients with heart failure (HF) and chronic obstructive pulmonary disease (COPD), respectively, concurrent with an episode of community-acquired pneumonia (CAP). We evaluated patients with pre-existing HF or COPD, beginning 30 days after CAP diagnosis, to determine if CAP had a prolonged impact on their underlying comorbidity.

**Methods:**

A retrospective matched-cohort design using US healthcare claims was employed. In each month of accrual, patients with HF or COPD who developed CAP (“CAP patients”) were matched (1:1, without replacement, on demographic and clinical profiles) to patients with HF or COPD who did not develop CAP (“comparison patients”). All patients were aged ≥40 years, and were pneumonia free during prior 1-year period. Exacerbation beginning 30 days after the CAP diagnosis and for the subsequent 1-year period were compared between CAP and comparison patients.

**Findings:**

38,010 (4·6%) HF patients and 48,703 (5·9%) COPD patients experienced a new CAP episode requiring hospitalization or outpatient care only, and were matched to comparison patients. In the HF subset, CAP patients were 47·2% more likely to experience an exacerbation vs patients without CAP (17·8% vs. 12·1%; p<0·001); in the COPD subset, CAP patients were 42·3% more likely to experience an exacerbation (16·2% vs. 11·4%; p<0·001).

**Conclusions:**

Our data provide evidence that CAP foreshadows a prolonged increase in risk of exacerbation of underlying HF or COPD in adults, and suggests a potential benefit to CAP prevention strategies.

## Introduction

Multiple studies have described an increased risk of incident cardiac events in adults hospitalized for pneumonia [1–4]. A meta-analysis reported that nearly 17% of hospitalized adults with pneumonia developed new onset congestive heart failure (HF), acute coronary syndrome, or cardiac arrhythmias within 30 days following admission [1]; most cardiac diagnoses occurred within the first week [5]. A five-fold excess early mortality has also been observed among hospitalized pneumonia patients who developed incident cardiac complications compared to hospitalized pneumonia patients without new onset cardiac events [5, 6]. Corrales-Medina reported a persistent increased risk for cardiovascular events, including new onset HF, following pneumonia which declines with time, but remains elevated for at least 5 years [7, 8].

Eurich also identified an increased risk of new onset HF over a 10 year follow up of adults with CAP [9].

Previously, we reported that non-immunocompromised adults with chronic heart and lung disease were at substantial increased risk for pneumonia compared to healthy adults with no comorbidities [10]. This observation, combined with evidence of short-term morbidity, increased risk for new onset HF [9], and excess early and late mortality in adults with comorbidity consequent and following pneumonia, led us to hypothesize that in adults with underlying HF or COPD, pneumonia has an adverse impact on the pre-existing condition subsequent to the acute phase of the pneumonia episode. Specifically, we evaluated whether adults with underlying HF or COPD were likely to suffer more frequent exacerbations of their underlying disease following an episode of pneumonia compared with matched HF and COPD patients who did not develop pneumonia.

## Methods

### Study design and data source

This study employed a retrospective matched-cohort design and data spanning January 2009 through June 2014 from an integrated US private healthcare claims repository—Truven Health Analytics MarketScan^®^ Commercial Claims and Encounters (CCAE) and Medicare Supplemental and Coordination of Benefits (MDCR) Databases (hereinafter, “MarketScan Database”). MarketScan Database comprises medical (i.e., facility and professional service) and outpatient pharmacy claims, and was de-identified prior to its release; its use was thus compliant with HIPAA Privacy Rule and federal guidance [11]. A detailed description of the study design, data sources, and study methods—including operational algorithms for identifying HF, COPD, and pneumonia—may be found online [Tables A, B, C, and D in S1 Appendix].

### Source population

The source population comprised all patients who, between January 2009 and June 2013, had evidence of HF or COPD defined as ≥1 hospitalization with a HF or COPD ICD-9-CM in the principal or secondary position on a claim, or ≥2 ambulatory encounters (separated by ≥30 days) with a HF or COPD diagnosis code and a filled prescription for a HF or COPD-related drug within 7 days. From the source population, two subsets of patients were identified: the first included all patients with evidence of HF (irrespective of COPD), and the second included all patients with evidence of COPD (irrespective of HF).

### Study population

Beginning with January 2010, all patients in the HF and COPD subsets of the source population who were aged ≥40 years, had continuous and comprehensive health benefits, evidence of HF or COPD, and no evidence of pneumonia during the prior year were identified. Incident episodes of community-acquired pneumonia (CAP) in these patients were identified monthly using ICD-9-CM diagnosis codes and NDC drug codes. CAP episodes were stratified by inpatient care vs. outpatient care only; subjects requiring inpatient care who died or spent >30 days in the hospital were excluded. Inpatient CAP episodes were identified based on encounters that resulted in diagnosis of pneumonia (any position) on facility inpatient claims; outpatient CAP episodes were identified based on encounters in the ambulatory setting that resulted in diagnosis of pneumonia, evidence of chest x-ray, and ≥1 dispense for antibiotic therapy, all within a period of 5 days. Qualifying subjects were designated “CAP patients” and assigned an index date defined as the date of the initial CAP encounter (or admission date for inpatients) +/- 30 days.

One comparison patient from the corresponding HF or COPD source population was matched to each CAP patient on index date, age (±1 year), sex, and selected markers for health status. Like CAP cases, comparison patients also were required to have continuous and comprehensive health benefits, evidence of HF or COPD, and no evidence of pneumonia during the year prior to the index date.

Matching was implemented for each CAP patient by identifying all candidate patients based on the aforementioned criteria, and randomly selecting one of the candidate comparison patients for inclusion in the study population. Markers of health status were ascertained during the 1-year period prior to the index date, and included the number of HF or COPD-related hospitalizations (0, 1, 2, ≥3) and hospital days (0, 1–5, 6–10, ≥11), number of HF or COPD-related ambulatory encounters (0, 1–4, 5–9, ≥10), and comorbidity profile (number of chronic conditions [1, 2, ≥3] and immunocompromising conditions [0, ≥1]). Once matched, both CAP and comparison patient were included in the study population and removed from the source population. The same process was repeated for each subsequent calendar month—using the pool of patients remaining in the source population after matching in prior months—ending with May 2013.

### Study measures

Study measures included exacerbations of HF and COPD and associated healthcare costs, and were ascertained beginning on the index date (i.e., 30 days after CAP event) and ending one year later or on the date of health plan disenrollment, whichever occurred first. Exacerbations were defined as a hospitalization or an emergency department encounter for the treatment of HF or COPD; hospitalizations were identified based on inpatient facility claims with a principal diagnosis, and emergency room visits with the condition of interest in any position. Initiation of digoxin therapy for HF and initiation of supplemental oxygen use for HF and COPD were considered as secondary study measures. Healthcare costs were tallied based on services rendered in the management of HF or COPD exacerbations, and were expressed in 2014 US dollars; costs from earlier years were adjusted using hospital and related services component of the Consumer Price Index.

### Data analysis

The adequacy of matching was evaluated by comparing the standardized difference (average difference between means measured in standard deviation units) between baseline characteristics (i.e., those not considered in the matching process; [Tables A and B in S3 Appendix]) of CAP and comparison patients [12, 13]; a standardized difference < 0·1 was assumed to indicate a negligible difference between CAP patients and matched comparison patients. We estimated the cumulative percentage of CAP and matched comparison patients who experienced an HF or COPD-related exacerbation by month stratified by inpatient vs. outpatient care only; we also examined differences by age (40–64 years, 65–74 years, and ≥75 years) and comorbidity status (presence or absence of immunocompromising conditions). The McNemar test was used to test for differences between groups. Incidence proportions were not adjusted for differential follow-up between CAP and comparison patients.

## Results

A total of 827,902 patients met our qualifying criteria for HF and were included in the HF source population. Among these patients, 38,010 (4·6%) experienced a new CAP episode requiring inpatient care or outpatient care only, and were matched to a comparison patient. For COPD, corresponding numbers were 828,677 patients in the source population and 48,703 (5·9%). Most of the matched CAP patients required hospitalization (HF, 87·0%; COPD, 83·1%), reflecting the stringent inclusion criteria for outpatient episodes of CAP. If only a diagnosis of CAP in the outpatient setting (and not concurrent x-ray and antimicrobial prescription) had been required to identify outpatient CAP episodes, about 50% of matched CAP patients would have required hospitalization (HF, 47%; COPD, 50%). A description of the number of subjects who met each qualifying criterion for inclusion in the source and study populations may be found online [Tables A and B in S2 Appendix].

Among hospitalized CAP patients and their matched counterparts, 79·8% in the HF subset (n = 26,389 pairs) were aged ≥65 years, and 62·5% were aged ≥75 years; in the COPD subset (n = 29,655 pairs), 73·2% were aged ≥65 years, and 49·1% were aged ≥75 years [Table 1]. Many patients had at least one other chronic comorbidity (41·0% of HF patients; 57·6% of COPD patients), and 44·8% of HF and 29·8% of COPD patients had an immunocompromising condition; 37·3% of subjects with HF and 25·1% of subjects with COPD had a HF- or COPD-related hospitalization within the prior year [Table 1]. In general, hospitalized CAP and matched comparison patients were well-paired on baseline characteristics not considered in the matching process; differences in mean or percentage values were noted for absence of atrial fibrillation in the hospitalized pneumonia group with HF and the use of supplemental oxygen in the hospitalized pneumonia group with either condition. Mean duration of follow-up was lower for CAP patients than comparison patients: HF, 9·3 months vs. 10·4 months (standard difference ≥0·1); COPD, 9·7 months vs. 10·6 months (standard difference ≥0·1). Characteristics of ambulatory CAP patients and their counterparts were largely comparable to each other, and are set forth in the online supplement [Tables A and B in S3 Appendix].

**Table 1:** Characteristics of hospitalized CAP patients and matched comparison patients in heart failure and chronic obstructive pulmonary disease populations*.

			Heart Failure		Chronic Obstructive Pulmonary Disease	
			CAP Patients (N = 33,068)	Comparison Patients (N = 33,068)	CAP Patients (N = 40,488)	Comparison Patients (N = 40,488)
Age (yrs), n (%)						
	40–49		659 (2.0)	648 (2.0)	872 (2.2)	855 (2.1)
	50–64		6,020 (18.2)	6,088 (18.4)	9,961 (24.6)	10,044 (24.8)
	65–74		5,705 (17.3)	5,670 (17.1)	9,766 (24.1)	9,755 (24.1)
	≥75		20,684 (62.5)	20,662 (62.5)	19,889 (49.1)	19,834 (49.0)
Sex, n (%)						
	Male		17,046 (51.5)	17,046 (51.5)	19,779 (48.9)	19,779 (48.9)
	Female		16,022 (48.5)	16,022 (48.5)	20,709 (51.1)	20,709 (51.1)
HF/COPD-Related Hospitalizations in Prior Year, n (%)^†^						
	0		20,741 (62.7)	20,741 (62.7)	30,336 (74.9)	30,336 (74.9)
	1		10,325 (31.2)	10,325 (31.2)	8,735 (21.6)	8,735 (21.6)
	2		1,512 (4.6)	1,512 (4.6)	1,076 (2.7)	1,076 (2.7)
	≥3		490 (1.5)	490 (1.5)	341 (0.8)	341 (0.8)
HF/COPD-Related Hospital Days in Prior Year, n (%)^†^						
	0		20,741 (62.7)	20,741 (62.7)	30,336 (74.9)	30,336 (74.9)
	1–5		6,380 (19.3)	6,380 (19.3)	5,793 (14.3)	5,793 (14.3)
	6–10		3,745 (11.3)	3,745 (11.3)	2,878 (7.1)	2,878 (7.1)
	≥11		2,202 (6.7)	2,202 (6.7)	1,481 (3.7)	1,481 (3.7)
HF/COPD-Related Outpatient Visits in Prior Year, n (%)^†^						
	0		3,991 (12.1)	3,991 (12.1)	2,878 (7.1)	2,878 (7.1)
	1–4		20,638 (62.4)	20,638 (62.4)	20,824 (51.4)	20,824 (51.4)
	5–9		4,798 (14.5)	4,798 (14.5)	6,180 (15.3)	6,180 (15.3)
	≥10		3,641 (11.0)	3,641 (11.0)	10,606 (26.2)	10,606 (26.2)
Comorbidity Profile						
	Chronic Condition^‡^, n (%)					
		1 Chronic Condition	4,692 (14.2)	4,692 (14.2)	5,090 (12.6)	5,090 (12.6)
		2 Chronic Conditions	7,623 (23.1)	7,623 (23.1)	11,269 (27.8)	11,269 (27.8)
		≥3 Chronic Conditions	5,931 (17.9)	5,931 (17.9)	12,060 (29.8)	12,060 (29.8)
	Immunocompromising Condition^§^		14,822 (44.8)	14,822 (44.8)	12,069 (29.8)	12,069 (29.8)

CAP: Community-acquired pneumonia; COPD: chronic obstructive pulmonary disease; HF: heart failure

*Patients identified between 1/2010 and 5/2013 within Truven Health Analytics MarketScan Commercial Claims and Encounters and Medicare Supplemental and Coordination of Benefits Databases

†Diagnosis of condition of interest in any position

‡Chronic heart disease, chronic lung disease, asthma, diabetes, alcoholism, chronic liver disease, smoker, Down's syndrome, neuromuscular/seizure disorder, short gestation/low birthweight, rheumatoid arthritis, Crohn's, lupus

§Asplenia, HIV, chronic renal failure, cochlear, immunosuppressants, congenital immunodeficiency, disease of white blood cells; patients with immunocompromising conditions are excluded from the chronic condition buckets

**Fig 1:**
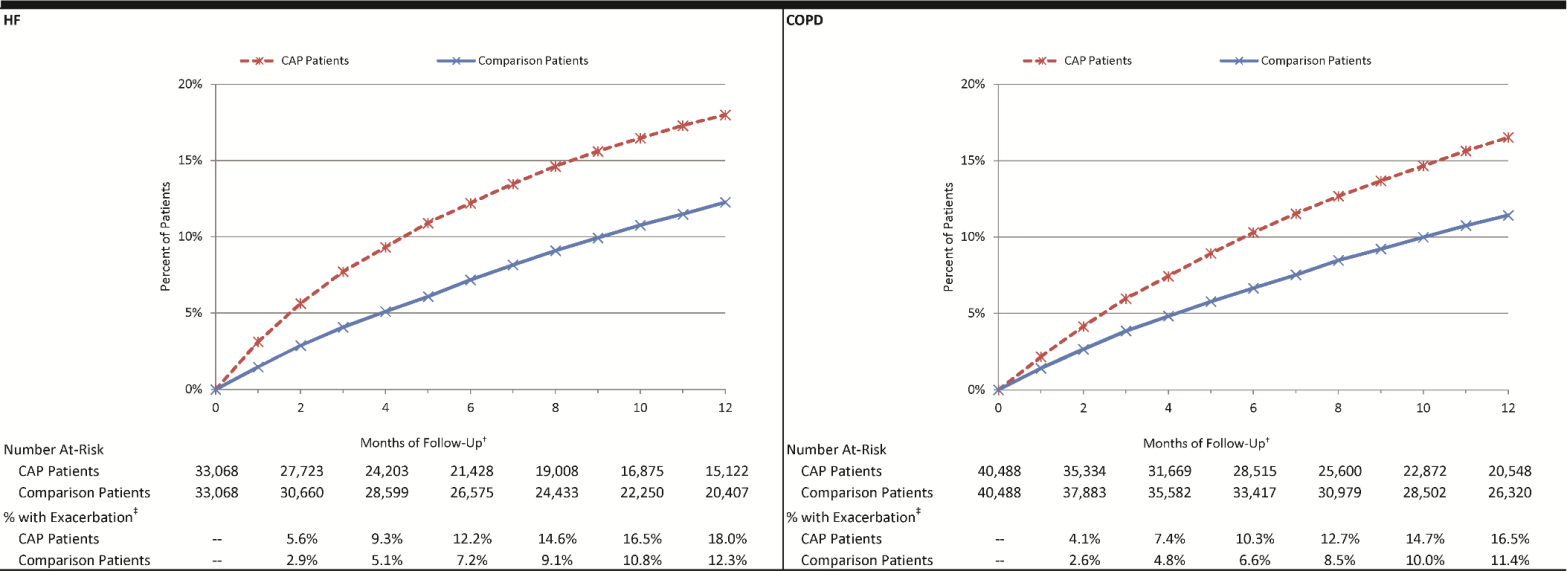
Cumulative percentage of hospitalized pneumonia patients and matched comparison patients who experienced an HF/COPD-related exacerbation*. CAP: Community-acquired pneumonia; COPD: chronic obstructive pulmonary disease; HF: heart failure. Exacerbation: Hospitalization with principal diagnosis of condition of interest, or emergency department visit with diagnosis of condition of interest in any position. *Patients identified between 1/2010 and 5/2013 within Truven Health Analytics MarketScan Commercial Claims and Encounters and Medicare Supplemental and Coordination of Benefits Databases. †Follow-up began 30 days after CAP diagnosis and ended 12 months later. ‡All comparisons p<0.001.

**Fig 2:**
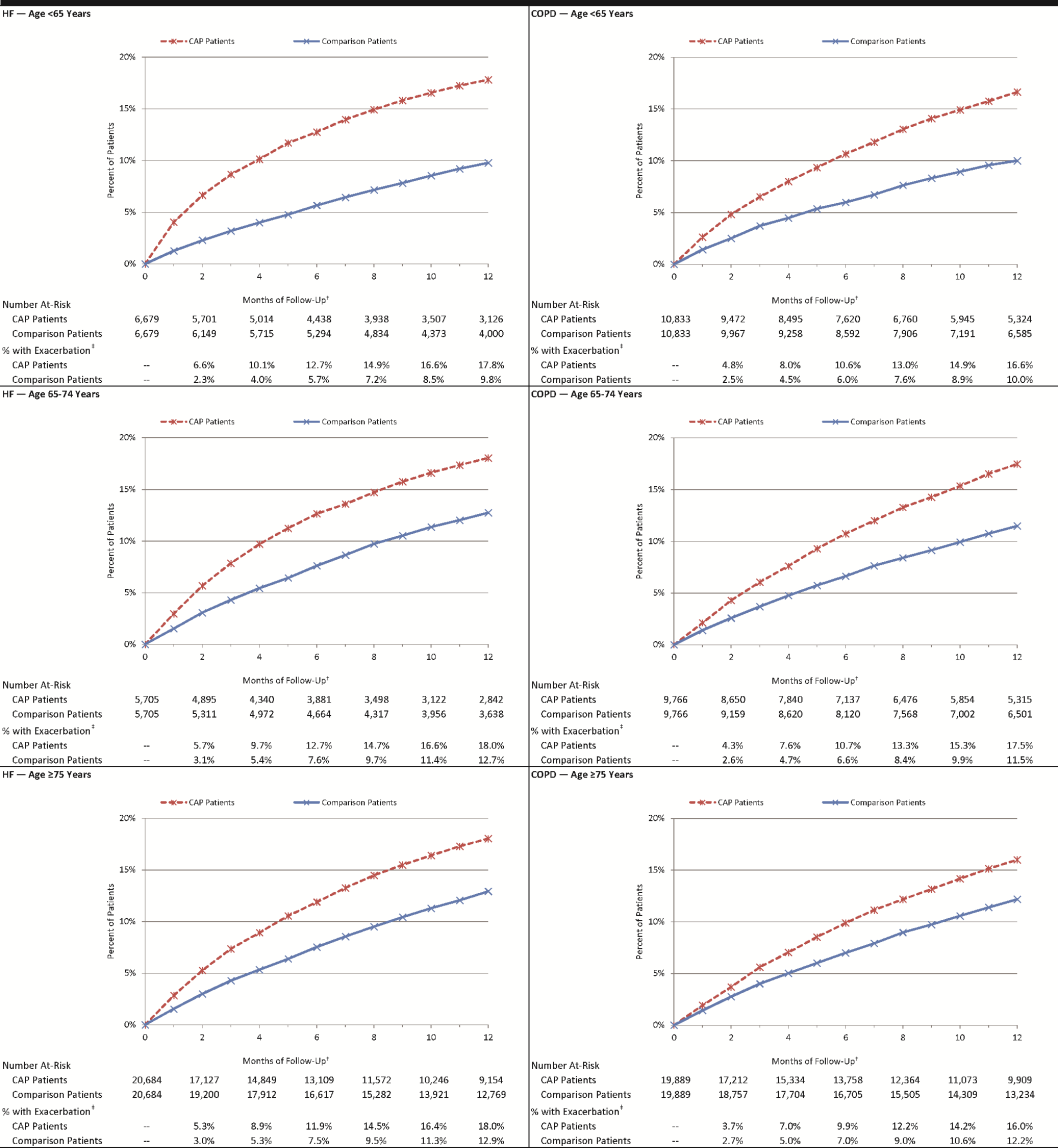
Cumulative percentage of hospitalized pneumonia patients and matched comparison patients who experienced an HF/COPD-related exacerbation, by age*. CAP: Community-acquired pneumonia; COPD: chronic obstructive pulmonary disease; HF: heart failure. Exacerbation: Hospitalization with principal diagnosis of condition of interest, or emergency department visit with diagnosis of condition of interest in any position. *Patients identified between 1/2010 and 5/2013 within Truven Health Analytics MarketScan Commercial Claims and Encounters and Medicare Supplemental and Coordination of Benefits Databases. †Follow-up began 30 days after CAP diagnosis and ended 12 months later. ‡All comparisons p<0.001.

**Fig 3:**
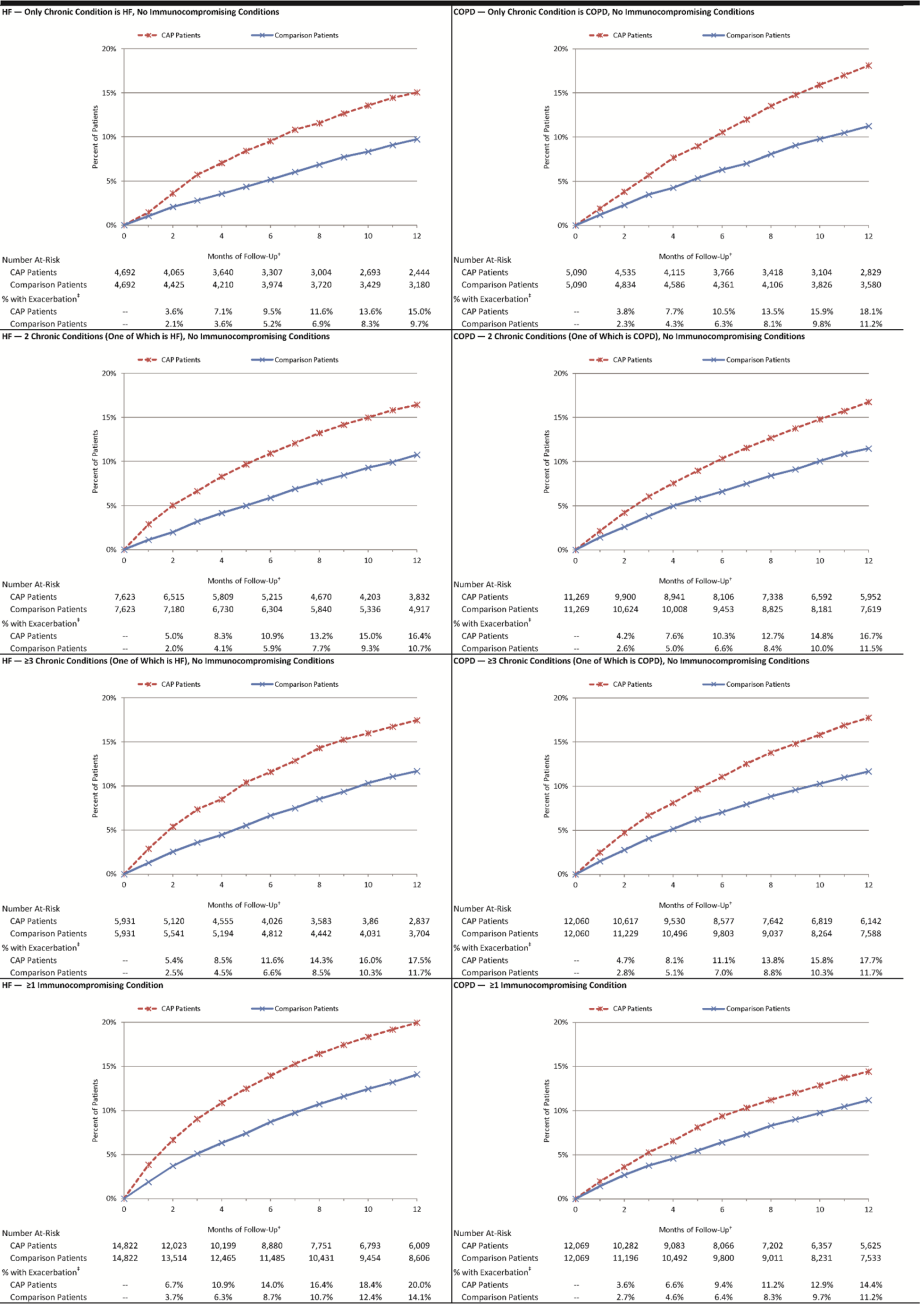
Cumulative percentage of hospitalized pneumonia patients and matched comparison patients who experienced an HF/COPD-related exacerbation, by comorbidity profile*. CAP: Community-acquired pneumonia; COPD: chronic obstructive pulmonary disease; HF: heart failure. Exacerbation: Hospitalization with principal diagnosis of condition of interest, or emergency department visit with diagnosis of condition of interest in any position. *Patients identified between 1/2010 and 5/2013 within Truven Health Analytics MarketScan Commercial Claims and Encounters and Medicare Supplemental and Coordination of Benefits Databases. †Follow-up began 30 days after CAP diagnosis and ended 12 months later. ‡All comparisons p<0.001.

Among the 33,068 matched pairs in the HF subset, the percentage of hospitalized CAP patients who experienced an exacerbation—defined as a HF-related hospitalization (principal diagnosis) or ED visit (diagnosis in any position)—by month 2 of follow-up was 5·6%, versus 2·9% among matched comparison patients (absolute difference = 2·8%, p<0·001). By month 12, corresponding percentages were 18·0% and 12·3% (absolute difference = 5·7%, p<0·001). Among 40,488 matched pairs in the COPD subset, the percentage of hospitalized CAP patients who experienced a COPD exacerbation by month 2 of follow-up was 4·1%, versus 2·6% among matched comparison patients (absolute difference = 1·5%, p<0.001) [Fig 1]. By month 12, corresponding percentages were 16·5% and 11·4% (absolute difference = 5·1%, p<0·001). Results for subgroups defined by age and comorbidity profiles [Figs 2 and 3], ambulatory CAP patients and their matched counterparts [Figs A, B, and C in S4 Appendix], and alternative measures of exacerbation (i.e., new digoxin use, new supplemental oxygen use) and duration of follow-up [Figs A, B, C, and D in S5 Appendix] also demonstrated excess exacerbations (age and comorbidity) or prescriptions (digoxin and oxygen) in CAP versus matched comparison patients.

In the HF subset, the mean cost of an exacerbation was $11,091 (ED visit: $559; hospitalization: $20,109) for all CAP and $10,893 (ED visit: $628; hospitalization: $20,327) for all comparison patients, and total 1-year costs were $97.7million versus $60·6 million, respectively. In the COPD subset, mean cost was $5,434 (ED visit: $594; hospitalization: $12,796) for all CAP and $5,278 (ED visit: $573; hospitalization: $13,097) for all comparison patients and total 1-year costs were $54·5 million versus $33·3 million, respectively, [Table A in S6 Appendix].

## Discussion

We compared HF- and COPD-specific exacerbations, beginning 30 days after diagnosis and at a time when the majority of acute signs and symptoms of CAP would have resolved, in patients ≥40 years of age with the pre-existing comorbidities of interest. We evaluated whether excess disease-specific exacerbations persisted beginning 30 days and extending for 13 months after the CAP diagnosis. We used a retrospective matched cohort design, stratified by age, number of comorbidities, and severity of underlying HF or COPD and compared the cumulative percentage of HF or COPD exacerbations in the two groups. We observed an overall increased risk of HF and COPD-related exacerbations in patients with pre-existing HF or COPD following an episode of CAP, across age and comorbidity subgroups, compared to a matched pneumonia-free cohort. We also observed an increased risk of exacerbations following an episode of CAP compared with the prior one-year period (when adjusted for differential follow-up). Our data provide additional evidence to support Corrales-Medina’s observation that pneumonia appears to be a risk factor for progression of underlying HF beyond the expected time for resolution of acute inflammatory signs [7] and Eurich’s finding of increased risk for new onset HF following CAP [9].

HF and COPD patients diagnosed with an episode of CAP were about 45% more likely to experience exacerbation of their pre-existing condition compared with HF and COPD patients who did not develop CAP. We observed a comparable increase in burden of HF or COPD disease exacerbation across all age cohorts studied (40–64, 65–74, and ≥75 years of age), as well as among HF or COPD patients who had no evidence of any other comorbidity. Although cardiac disease is common in adults with COPD and is recognized to be adversely impacted by CAP, we observed increased hospitalizations and ER visits in individuals with COPD as their only comorbidity. This supports the hypothesis that pneumonia or host response impacts lung function negatively as well.

Treatment cost of the HF or COPD exacerbation was also higher in patients who had CAP. While the difference in mean cost of an exacerbation encounter was small between patients with and without a CAP episode, when aggregated across the study population, it was quite substantial. The excess cost burden of pneumonia in the presence of underlying comorbidities has been well documented in adults of all ages [14–18]. More frequent HF and COPD exacerbations could partially explain previous finding of increased pneumonia cost burden in adults with comorbidities.

The potential biological mechanism leading to HF or COPD progression following an acute pneumonia episode have not yet been precisely defined. Speculation that the inflammatory response is, in part, harmful underlies the current thinking as prior studies have identified inflammation as a risk factor for development of coronary artery disease [19, 20]. In support of the role of inflammation, levels of interleukin 6 measured at the time of hospital discharge in patients with pneumonia identify patients at greater risk of cardiovascular related mortality within a one year follow-up [21].

For both the HF and COPD populations, there was an increased cumulative percentage of HF or COPD exacerbations among CAP patients compared with matched counterparts at all time points. The interval differences (the change from 0 to 2 months, from 2 to 4 months, and so on), for both HF and COPD, in combined hospitalization and ER visits was greater in those recovering from CAP compared to matched controls suggesting that the excess burden persisted for most, if not all, of the study period. These findings are consistent with Corrales-Medina’s observation that the relative risk for cardiovascular events was significantly increased in each year during a ten year follow-up of previously healthy individuals with pneumonia. In addition, we also assessed whether patients with underlying HF received an increased number of digoxin prescriptions, and whether patients with HF or COPD received an increased number of supplemental oxygen prescriptions, as an alternative marker of HF or COPD progression. The proportion of CAP patients prescribed digoxin (HF patients) or supplemental oxygen (HF or COPD patients) increased more rapidly in the immediate post CAP window, and the magnitude of the difference between CAP and comparison patients was greatest for the first 6 months [Figs A, B, C, and D in S5 Appendix].

These observations may suggest two different mechanisms, one relevant to early cardiopulmonary events (such as demand ischemia and lung inflammation), and a second mechanism that alters the subsequent rate of progression of the underlying comorbidity. Brown and colleagues [22] have proposed a specific mechanism where pneumococcal infection could result in long term damage to the myocardium with resulting dysfunction. However, published data linking pneumonia with cardiovascular disease is primarily based on patients with ‘all cause’ pneumonia in whom a specific pathogen has not been identified. Increased myocardial infarction following influenza outbreaks and higher cardiovascular mortality during influenza epidemics in patients with pre-existing coronary artery disease have been reported as well [23–26].

The limitations of our analysis are related to use of an insurance claims database to identify and match subjects with and without CAP. We also recognize that although subjects were closely matched on demographic features, presence of comorbidities, and hospitalizations for the condition of interest in the prior year, our analysis of characteristics did reveal small differences in oral steroid use (COPD), history of atrial fibrillation (HF), and use of supplemental oxygen (HF and COPD) [Tables A and B in S3 Appendix]. As well, the use of claims data does not allow us to match on smoking status. It would have been preferable to match using precise physiological measures of disease progression such as ejection fraction or six minute walk; however, such measures are not available from a claims database. Study results were not adjusted for differential follow-up between CAP patients and comparison patients as we could not differentiate health plan disenrollment due to death versus other reasons. We suspect, however, that a disproportionate percentage of disenrollment in the CAP group (relative to the comparison group) was due to death, given the advanced age of the study population and correspondingly high case-fatality rates for CAP [27]. Study findings likely represent an underestimate of the excess exacerbations observed in those with known HF or COPD.

## Conclusion

In summary, our findings add to those that link CAP with longer-term adverse impacts on cardiac and pulmonary disease. Moreover, we observed an increased risk of exacerbations in those 40–64 years of age as well as older subjects, among those with HF alone or COPD alone, and among those with multiple comorbidities [10]. These data, if confirmed, suggest successful pneumonia prevention in cardiac and pulmonary disease patients could alter longer term outcomes. Alternatively, addressing the underlying mechanisms, such as chronic inflammation and/or platelet activation, could potentially alter the frequency of exacerbations of HF or COPD [20]. Smoking cessation and vaccination against influenza and pneumococcal disease are currently recommended for the prevention of CAP [28]. Several studies have reported decreases in cardiovascular mortality following influenza vaccination in high risk patients or linkage between influenza vaccination and decreased hospitalization with CAP [29, 30]. In one recent study [31], a pathogen was identified in only 38% of pneumonia cases and only 11% of cases (6% influenza virus; and 5% *Streptococcus pneumoniae*) were due to pathogens for which there are current vaccine programs. The challenge will be identifying additional effective prevention strategies.

## Supporting information

S1 Appendix **Table A.** Diagnosis, procedure, and drug codes for algorithms identifying source population, **Table B.** Diagnosis, procedure, and drug codes for algorithms identifying baseline chronic conditions, **Table C.** Diagnosis, procedure, and drug codes for algorithms identifying baseline immunocompromising conditions, **Table D.** Diagnosis, procedure, and drug codes for algorithms identifying pneumonia.
(DOC)

S2 Appendix **Table A.** Selection of source populations, **Table B.** Selection of study populations.
(DOC)

S3 Appendix **Table A.** Characteristics of CAP patients and matched comparison patients in heart failure population, **Table B.** Characteristics of CAP patients and matched comparison patients in chronic obstructive pulmonary disease population.
(DOC)

S4 Appendix **Fig A.** Cumulative percentage of ambulatory CAP patients and matched comparison patients who experienced an HF/COPD-related exacerbation, **Fig B.** Cumulative percentage of ambulatory CAP patients and matched comparison patients who experienced an HF/COPD-related exacerbation, by age, **Fig C.** Cumulative percentage of ambulatory CAP patients and matched comparison patients who experienced an HF/COPD-related exacerbation, by comorbidity profile.
(DOC)

S5 Appendix **Fig A.** Cumulative percentage of hospitalized CAP patients and matched comparison patients who experienced an HF/COPD-related alternative exacerbation, **Fig B.** Cumulative percentage of ambulatory CAP patients and matched comparison patients who experienced an HF/COPD-related alternative exacerbation, **Fig C.** Cumulative percentage of hospitalized CAP patients and matched comparison patients who experienced an HF/COPD-related exacerbation, **Fig D.** Cumulative percentage of ambulatory CAP patients and matched comparison patients who experienced an HF/COPD-related exacerbation.
(DOC)

S6 Appendix **Table A.** Economic costs attributable to exacerbation encounters.
(DOC)
