# Supplementary material for: Risk of exacerbation following pneumonia in adults with heart failure or chronic obstructive pulmonary disease
Source: PLoS One. 2017 Oct 13;12(10):e0184877. doi: 10.1371/journal.pone.0184877 (PMC5640217; doi:10.1371/journal.pone.0184877)

ONLINE SUPPLEMENT 1 – APPENDIX:

**DATA SOURCE DESCRIPTION**

This study employed a retrospective matched-cohort design and data from a large integrated US private healthcare claims repository—Truven Health Analytics MarketScan® Commercial Claims and Encounters (CCAE) and Medicare Supplemental and Coordination of Benefits (MDCR) Databases (hereinafter, the “MarketScan Database”) spanning January 2009 through June 2014. The CCAE Database contains healthcare claims information for employees of large, self-insured corporations and their dependents, along with data from a few commercial health plans. The MDCR Database contains healthcare claims information (including Medicare and employer-sponsored plans) for Medicare-eligible retirees, and includes only plans where both the Medicare-paid amounts and employer-paid amounts are available. Participating plans are located throughout the US, and provide health benefits to >15 million persons annually.

Available data from each facility and professional-service claim include dates and places of service, diagnoses, procedures performed/services rendered, and quantity of services (professional-service claims only)**.** Available data from each outpatient pharmacy claim include the drug (class) dispensed, dispensing date, quantity dispensed, and number of days supplied. Medical and pharmacy claims also include amounts paid (i.e., reimbursed) by health plans and patients for healthcare services rendered. Selected demographic and eligibility information (including age, sex, geographic region of residence, dates of plan eligibility) is available for all health plan enrollees in the databases. All data can be arrayed to provide a detailed chronology of medical and pharmacy services used by each plan member over time.

The data extract was de-identified prior to its release to study investigators, and thus its use for health services research is compliant with the HIPAA Privacy Rule and federal guidance on Public Welfare and the Protection of Human Subjects.

**ONLINE SUPPLEMENT 1 – APPENDIX:**

**ALGORITHMS AND CODES FOR IDENTIFYING CONDITIONS OF INTEREST**


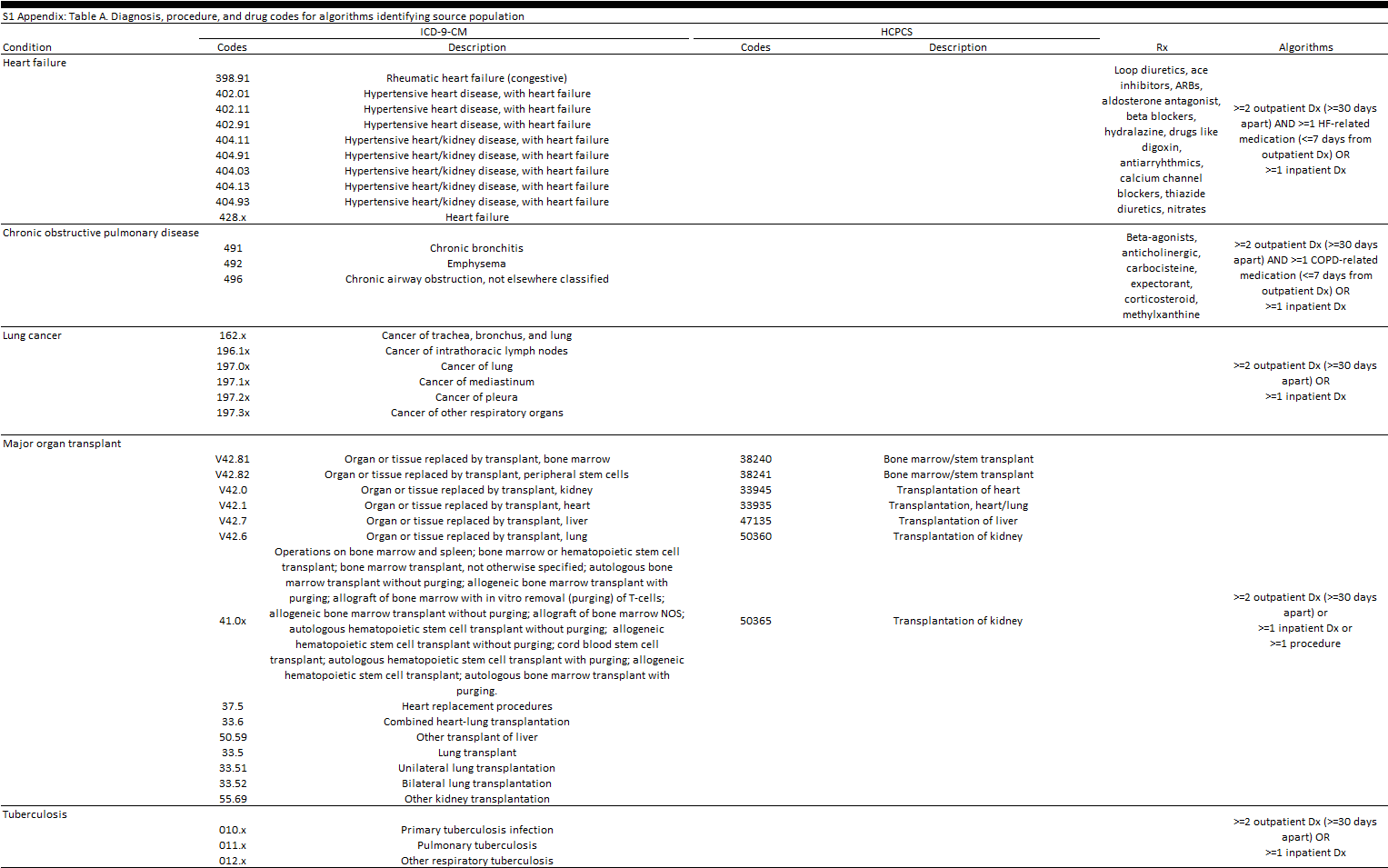


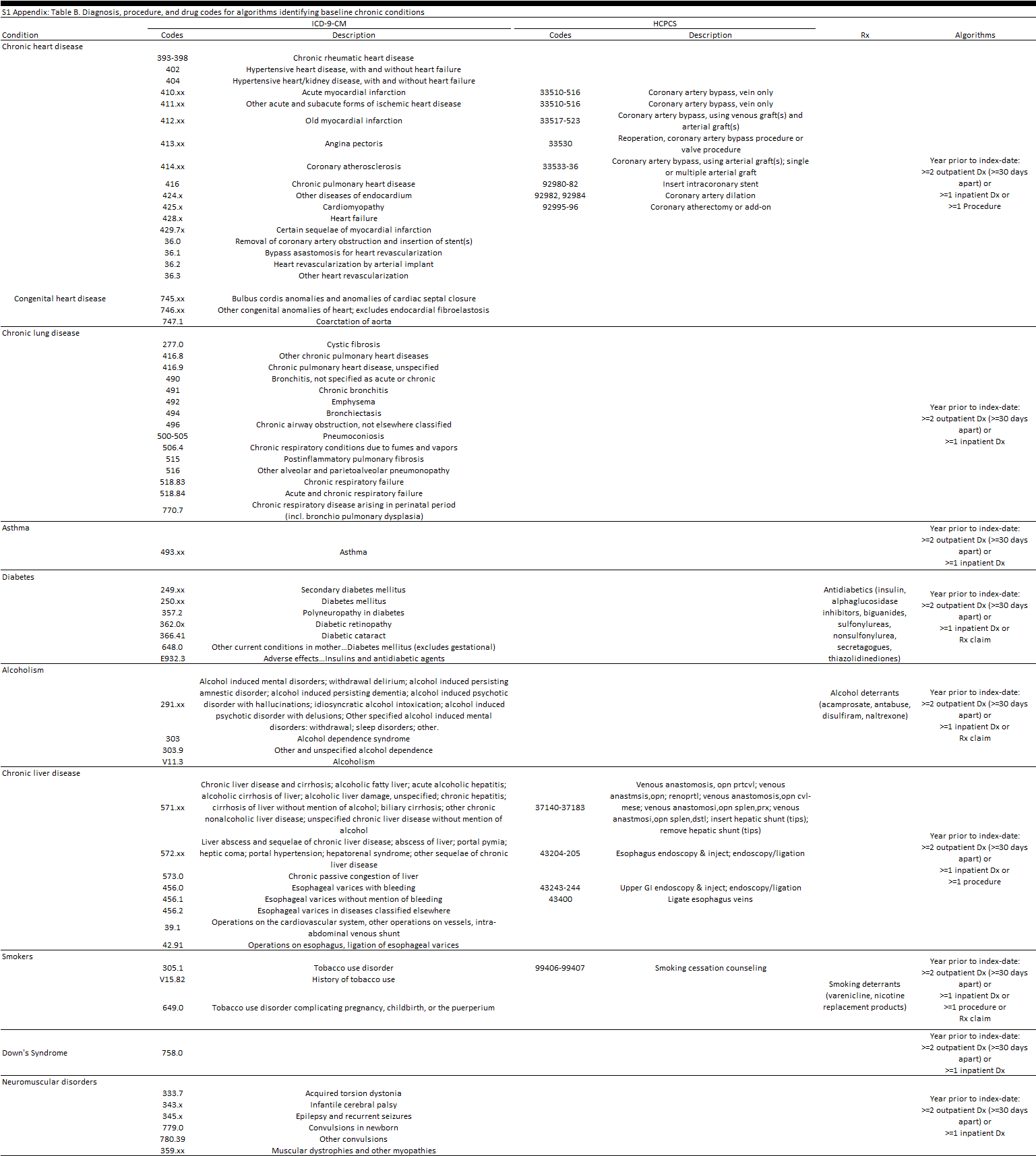

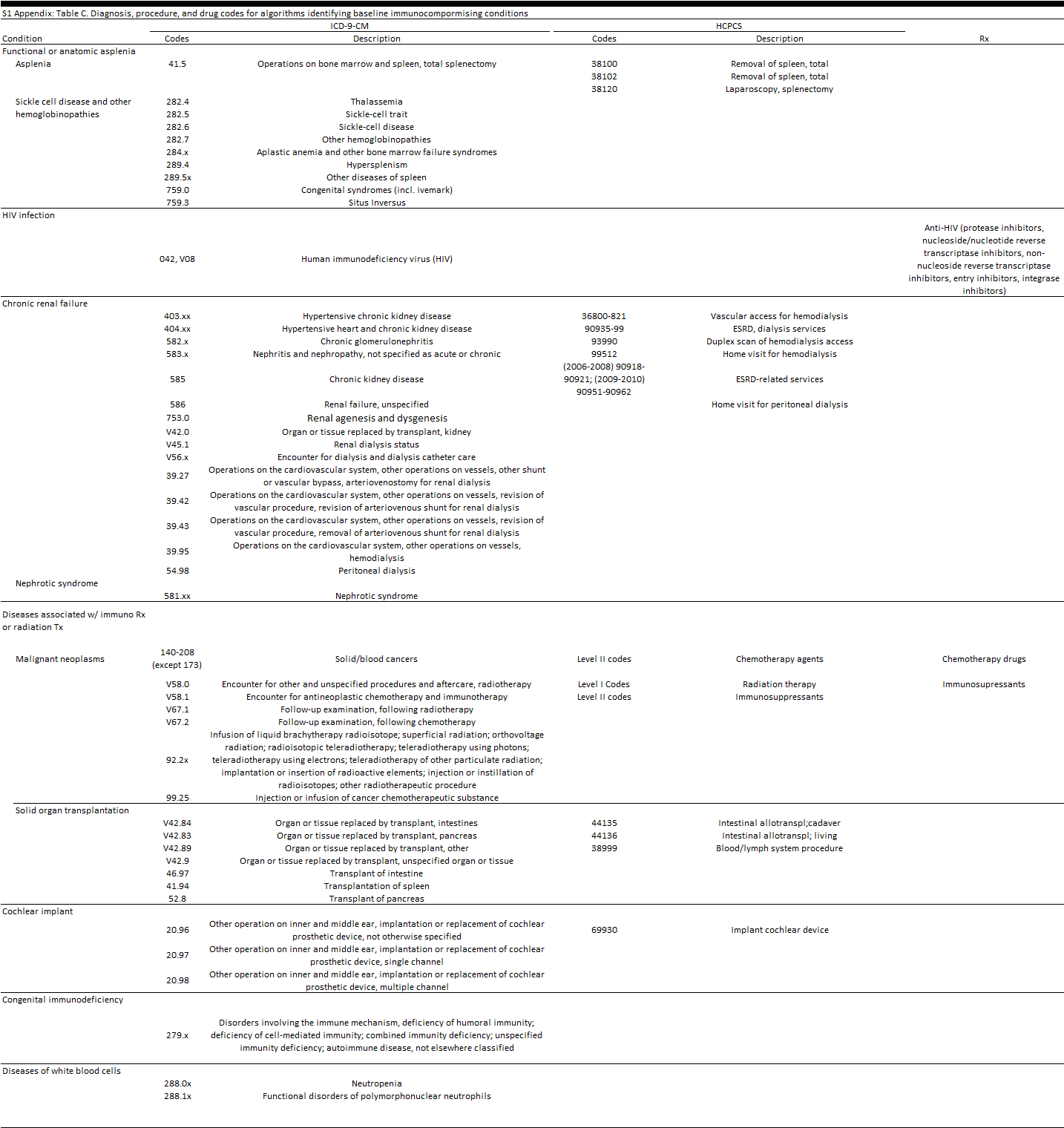


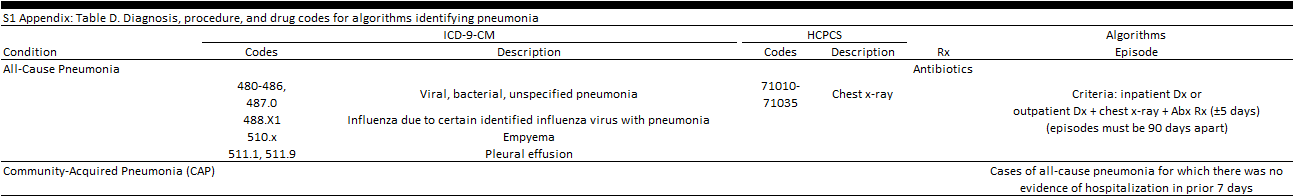

Supplement: S1 Appendix — Table A. Diagnosis, procedure, and drug codes for algorithms identifying source population, Table B. Diagnosis, procedure, and drug codes for algorithms identifying baseline chronic conditions, Table C. Diagnosis, procedure, and drug codes for algorithms identifying baseline immunocompromising conditions, Table D. Diagnosis, procedure, and drug codes for algorithms identifying pneumonia. (DOC) [file pone.0184877.s001.doc]
