# Supplementary figures and images for: Risk of exacerbation following pneumonia in adults with heart failure or chronic obstructive pulmonary disease
Source: PLoS One. 2017 Oct 13;12(10):e0184877. doi: 10.1371/journal.pone.0184877 (PMC5640217; doi:10.1371/journal.pone.0184877)

ONLINE SUPPLEMENT 2 – APPENDIX:

SELECTION ON SOURCE AND STUDY POPULATIONS


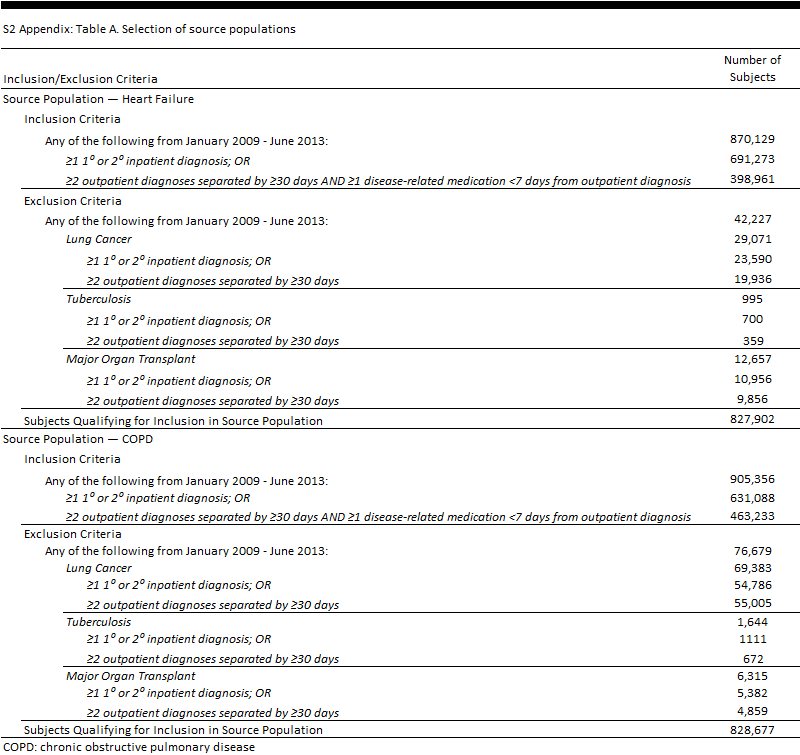


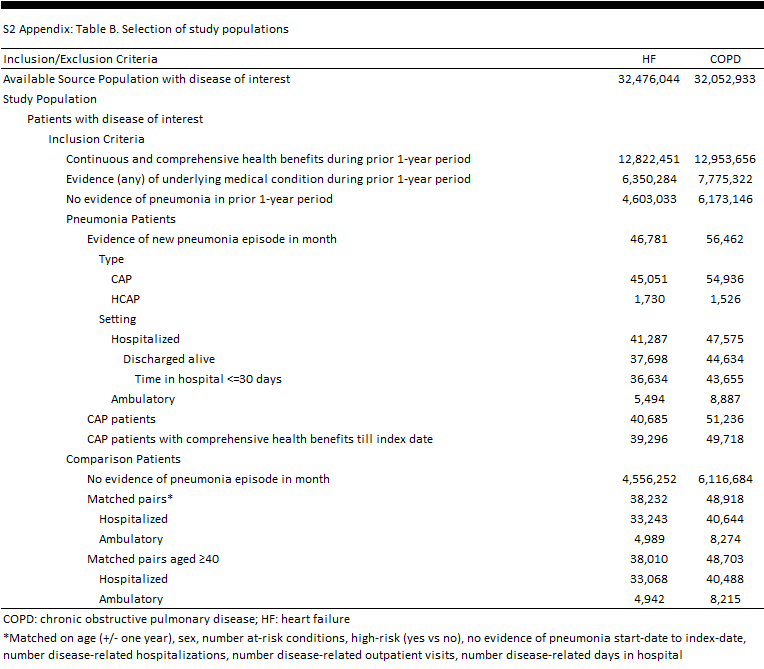

Supplement: S2 Appendix — Table A. Selection of source populations, Table B. Selection of study populations. (DOC) [file pone.0184877.s002.doc]

ONLINE SUPPLEMENT 3 – APPENDIX:

CHARACTERISTICS OF STUDY POPULATIONS
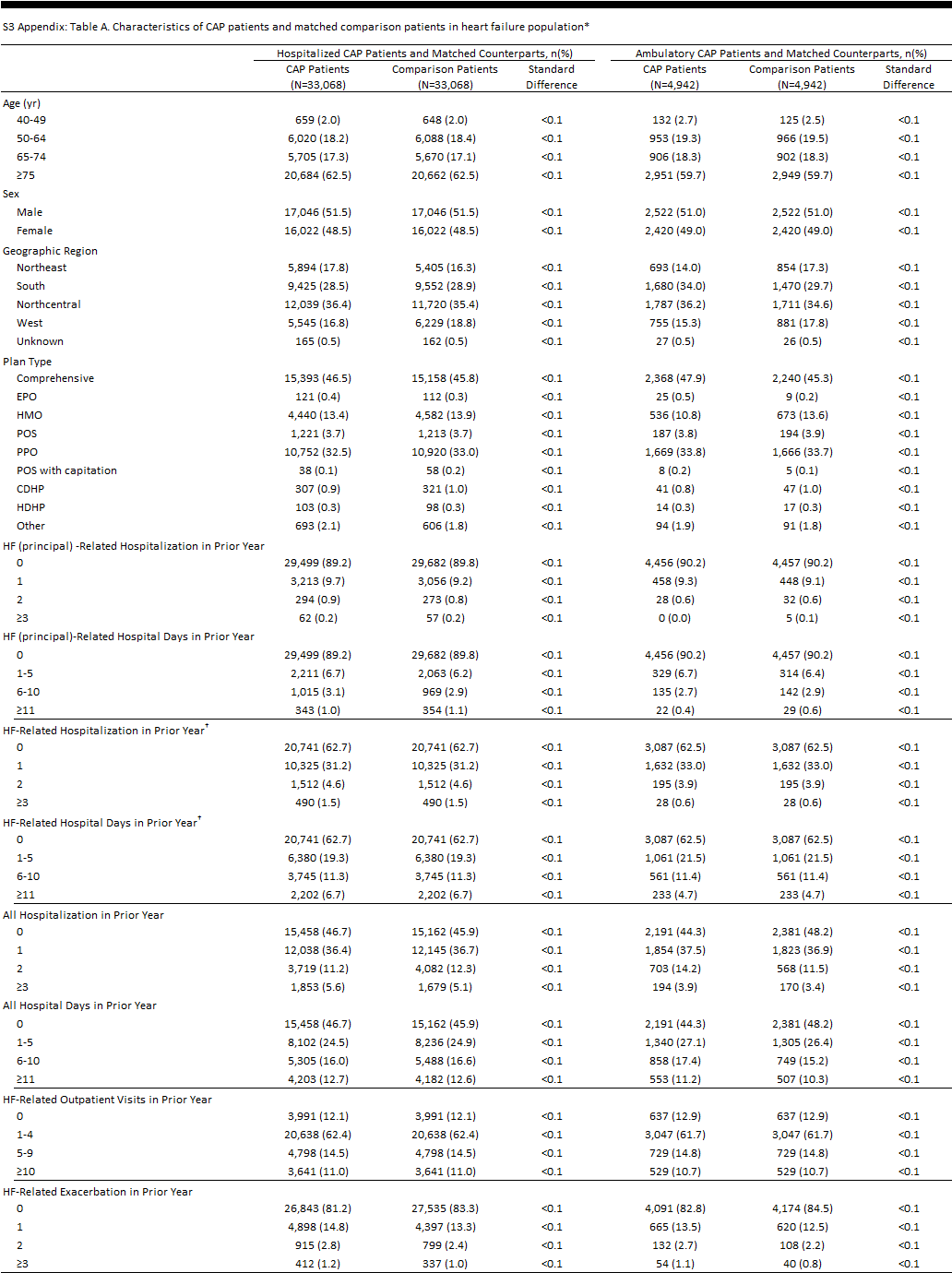

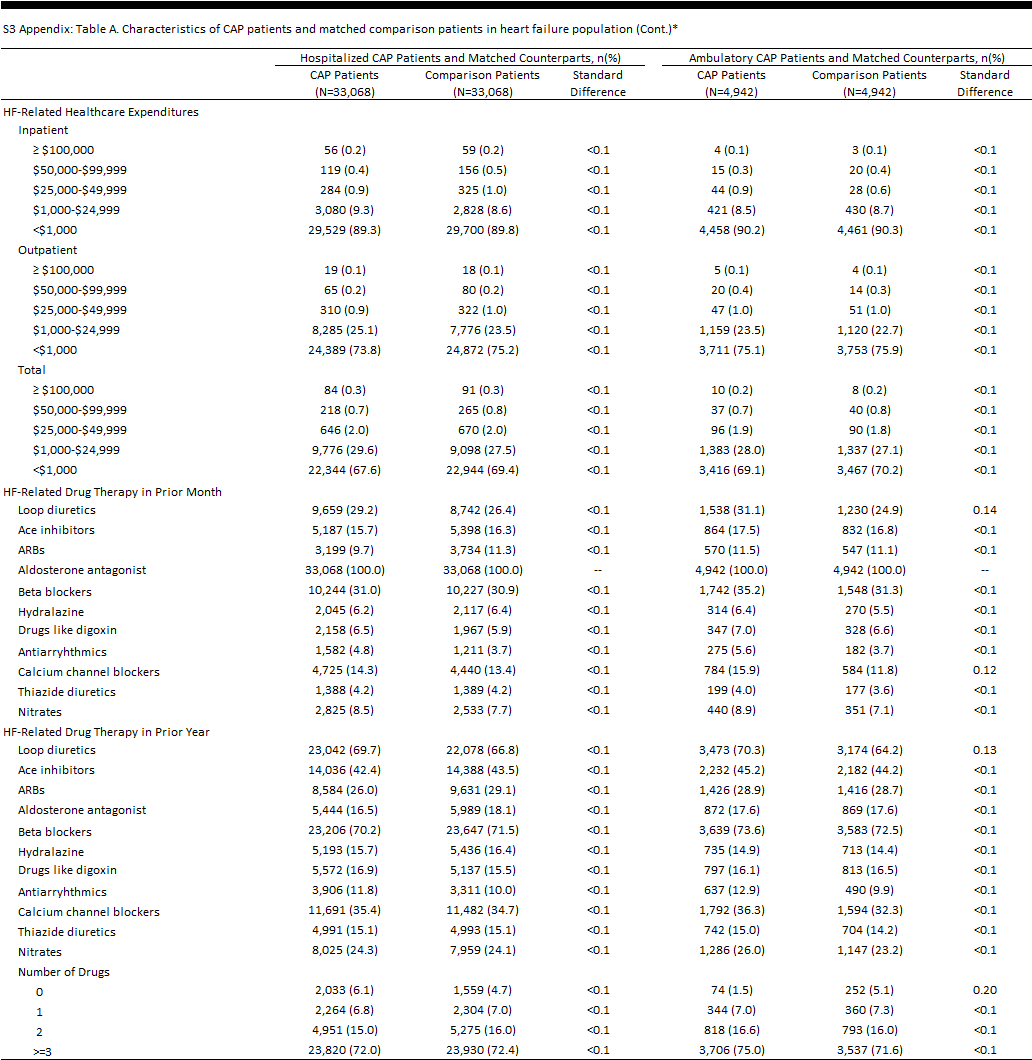


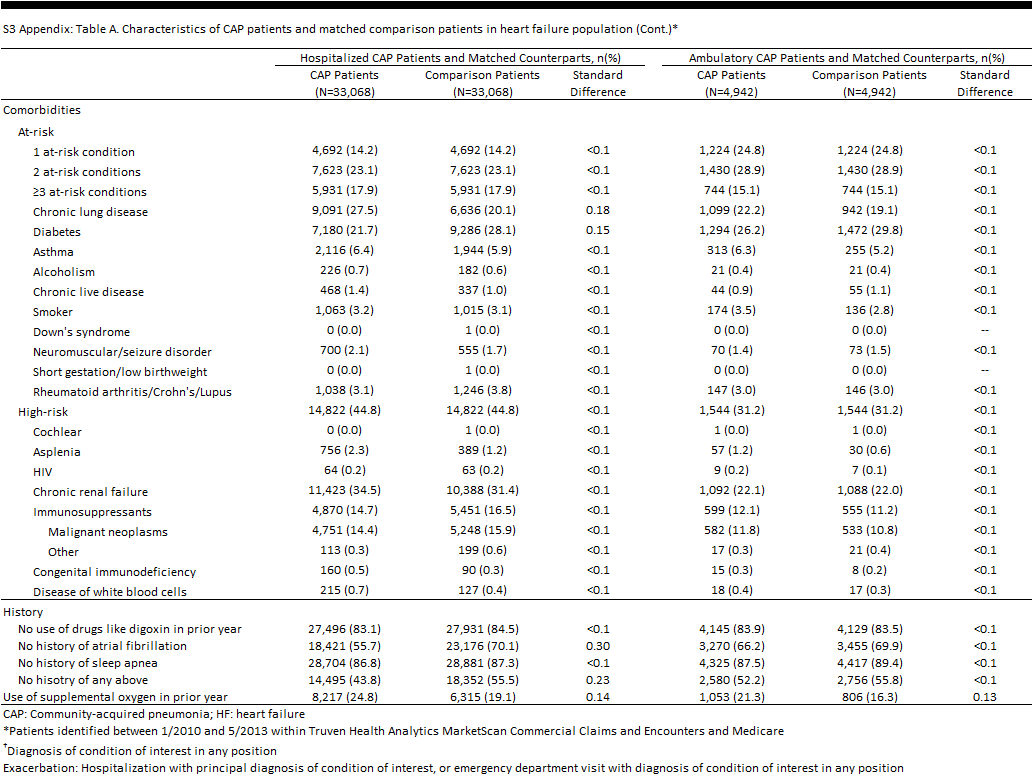

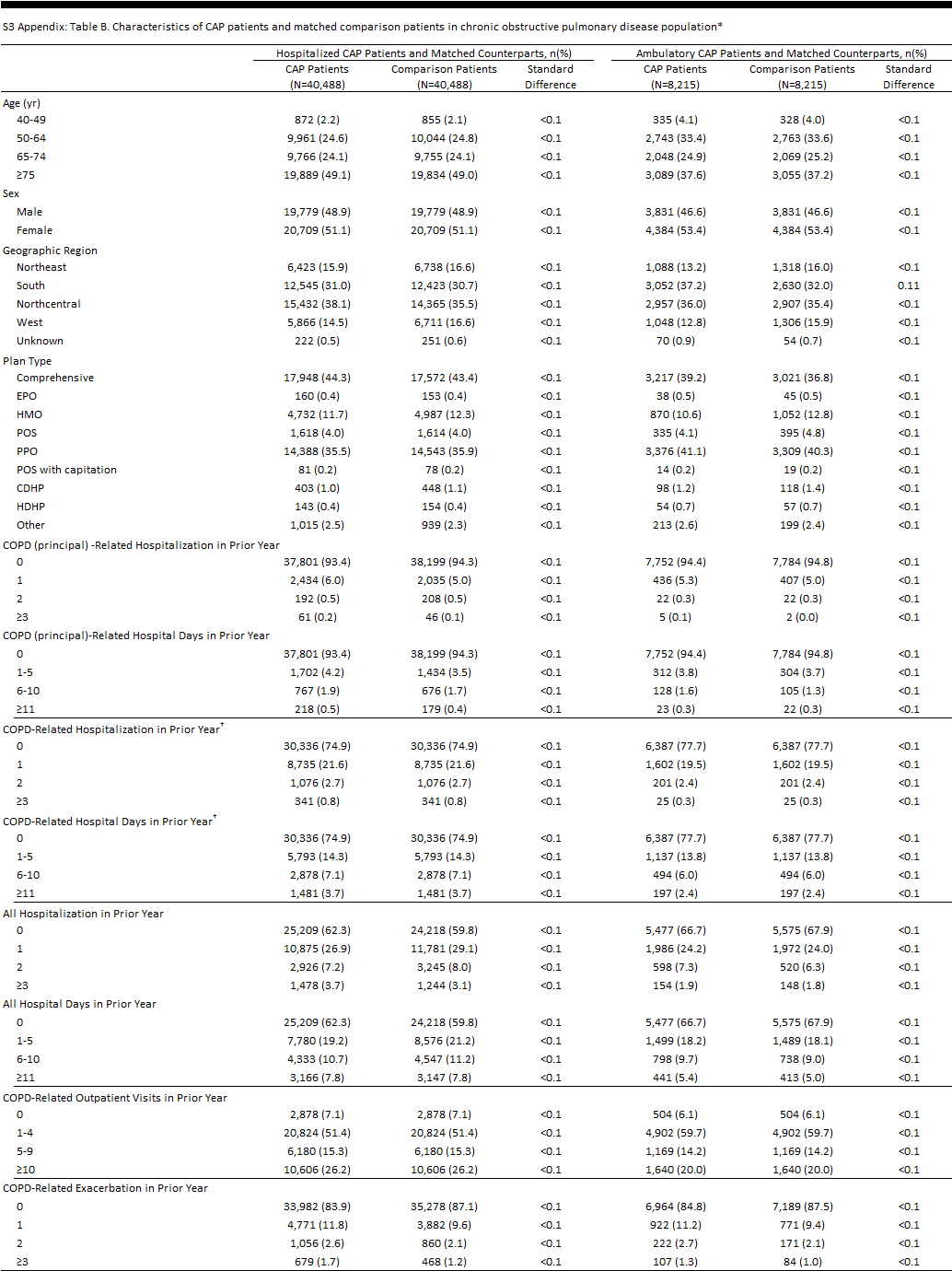

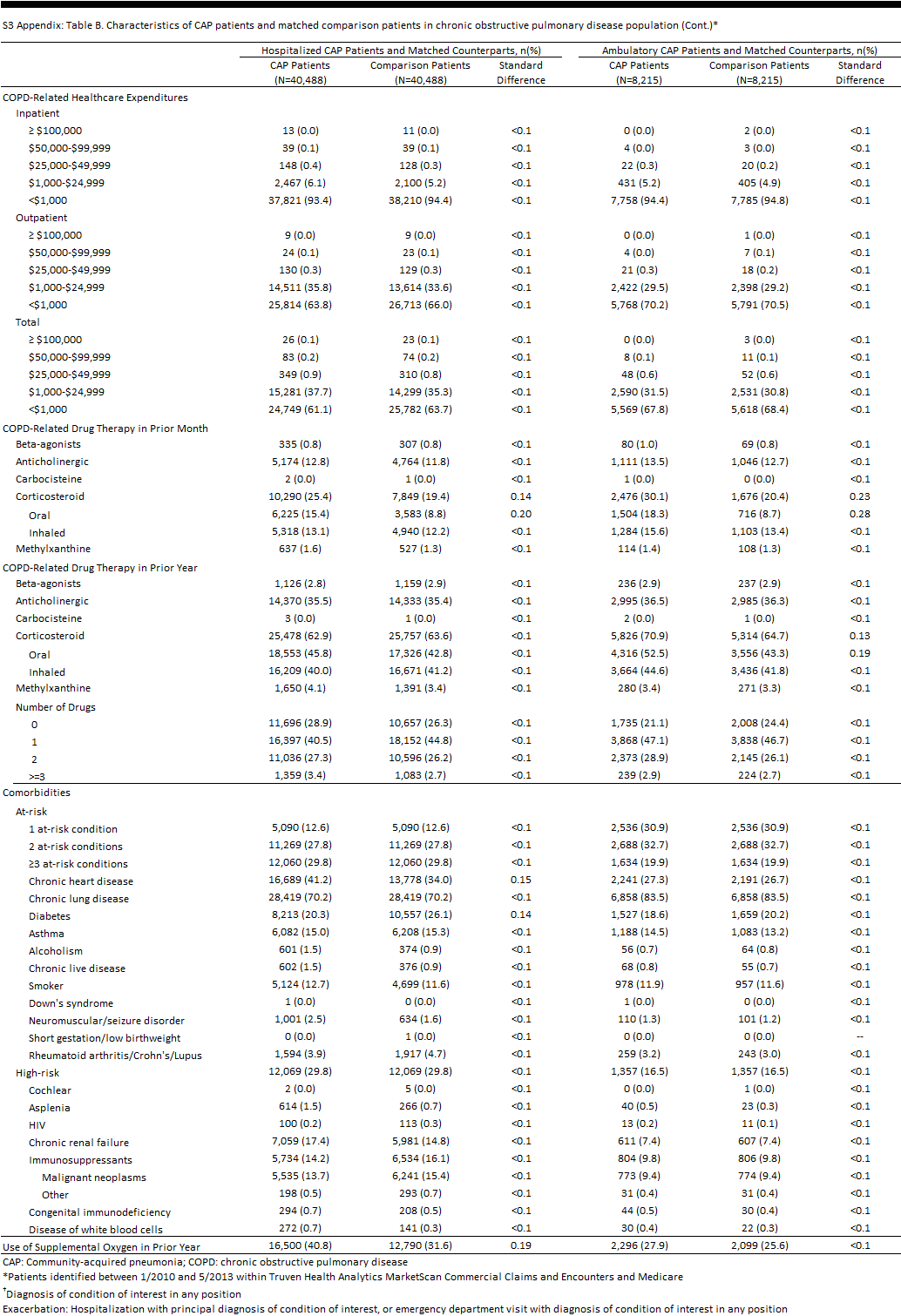

Supplement: S3 Appendix — Table A. Characteristics of CAP patients and matched comparison patients in heart failure population, Table B. Characteristics of CAP patients and matched comparison patients in chronic obstructive pulmonary disease population. (DOC) [file pone.0184877.s003.doc]

ONLINE SUPPLEMENT 5 – APPENDIX:

**RESULTS FROM ANALYSES OF DIGOXIN USE, OXYGEN USE, AND 2-YEAR FOLLOW-UP**


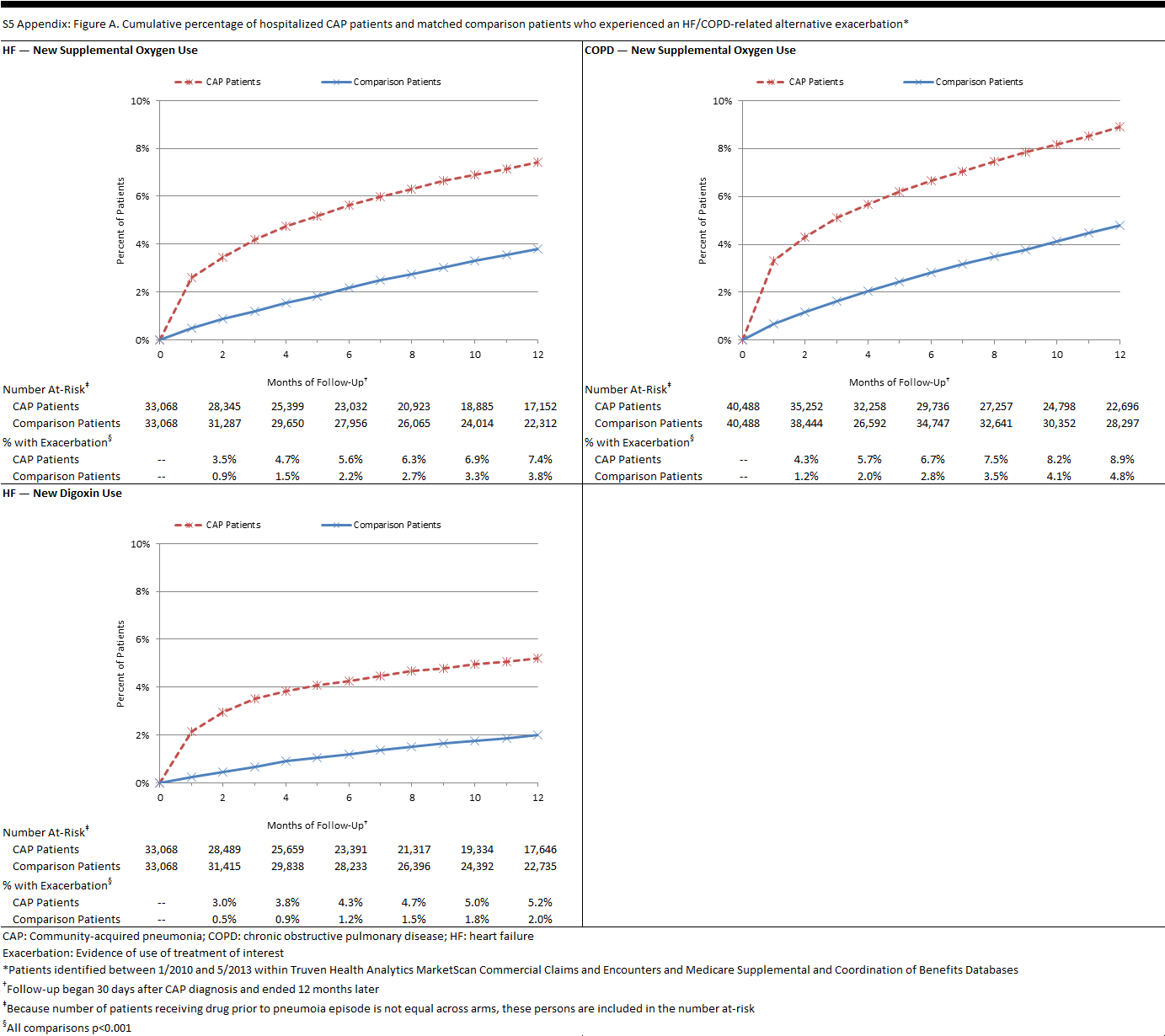


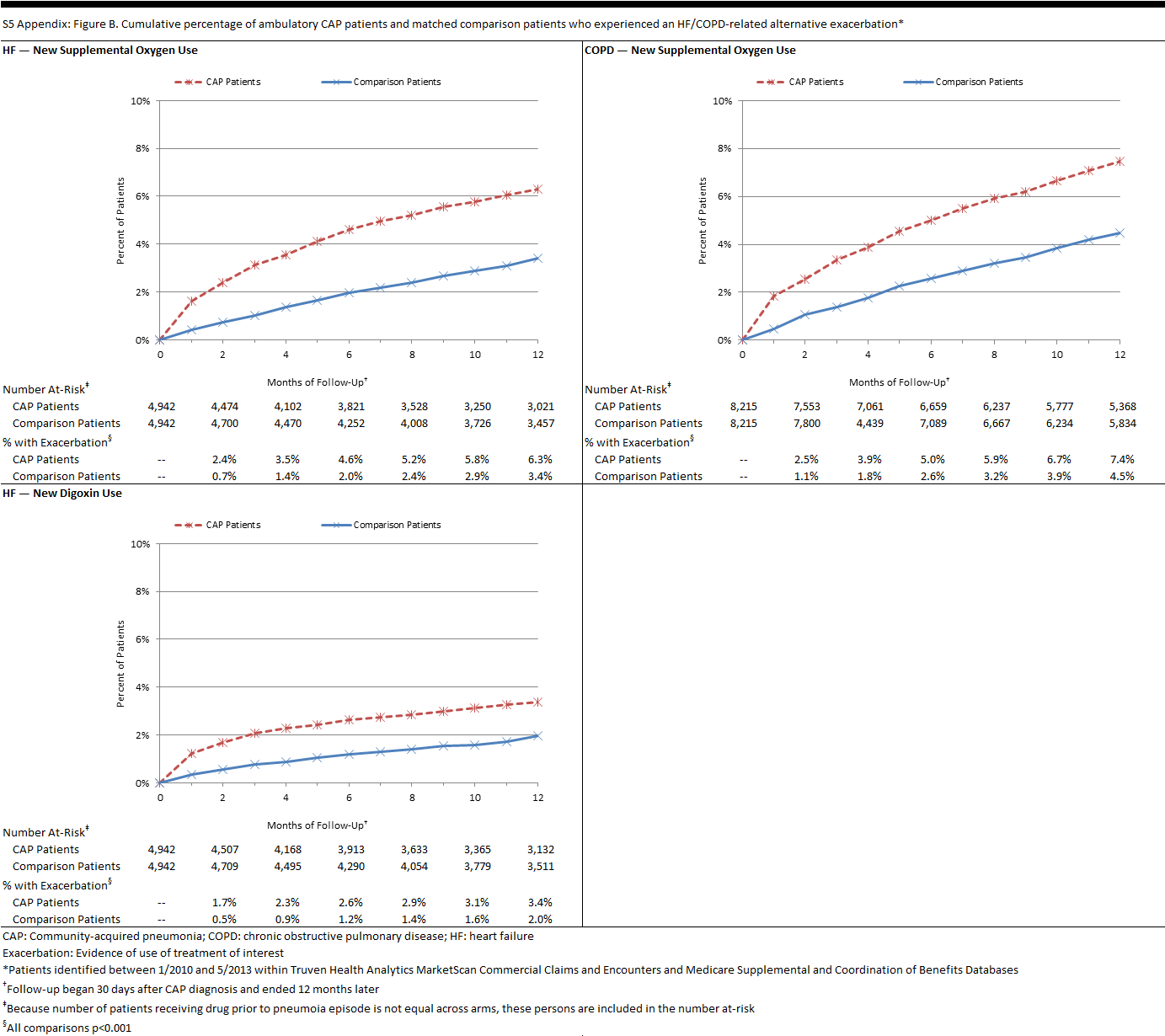


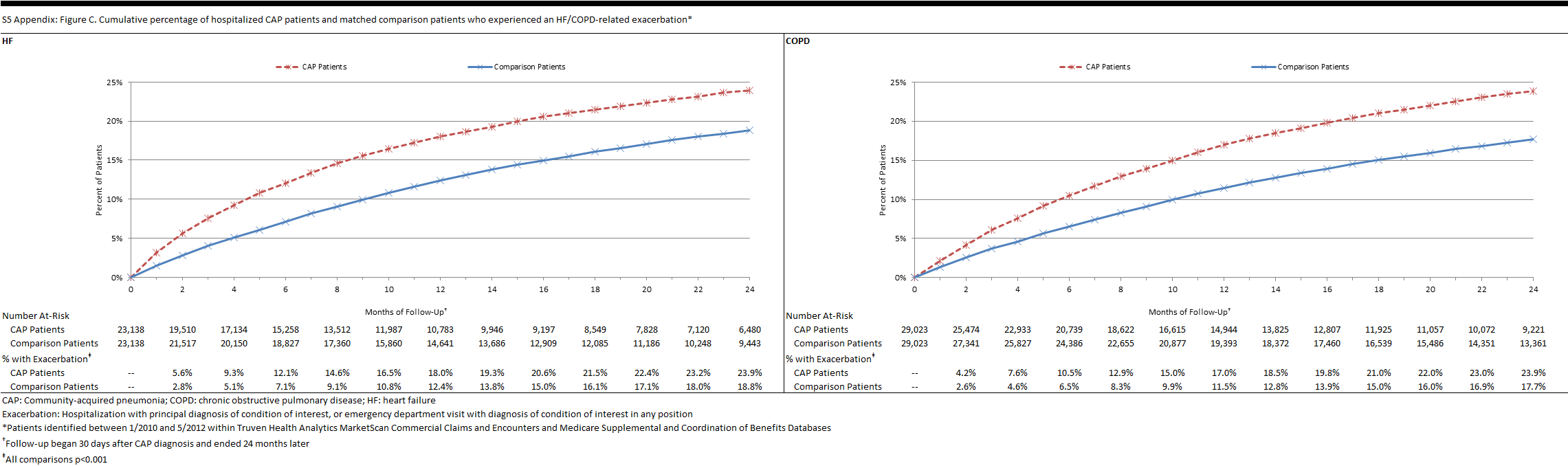


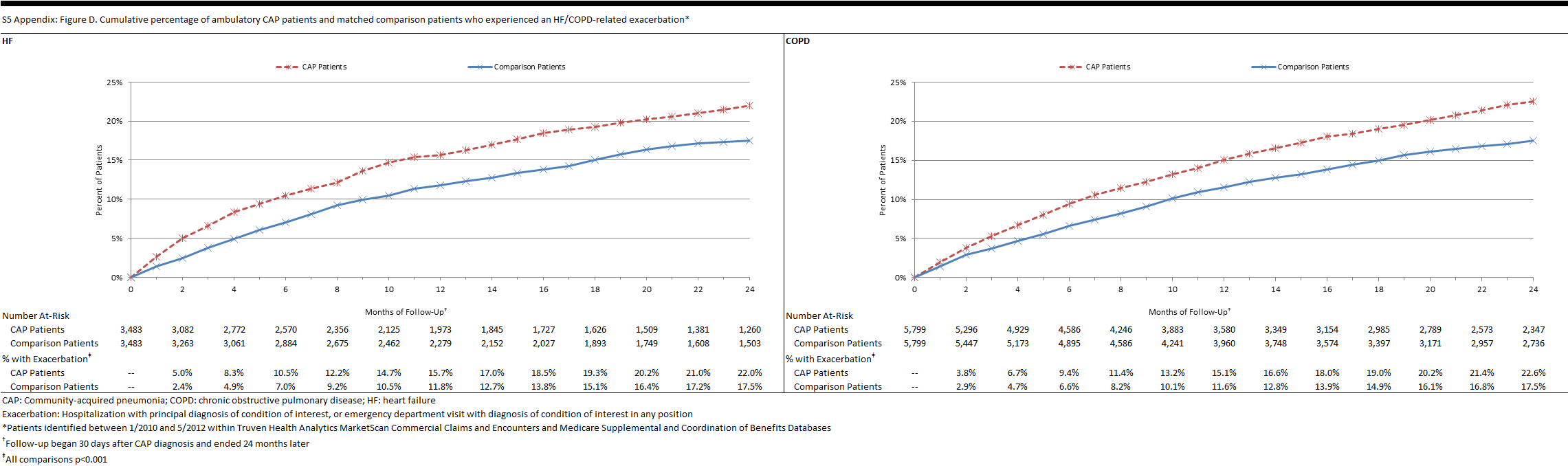

Supplement: S5 Appendix — Fig A. Cumulative percentage of hospitalized CAP patients and matched comparison patients who experienced an HF/COPD-related alternative exacerbation, Fig B. Cumulative percentage of ambulatory CAP patients and matched comparison patients who experienced an HF/COPD-related alternative exacerbation, Fig C. Cumulative percentage of hospitalized CAP patients and matched comparison patients who experienced an HF/COPD-related exacerbation, Fig D. Cumulative percentage of ambulatory CAP patients and matched comparison patients who experienced an HF/COPD-related exacerbation. (DOC) [file pone.0184877.s005.doc]

ONLINE SUPPLEMENT 6 – APPENDIX:

**RESULTS FROM ANALYSES OF ECONOMIC COSTS**


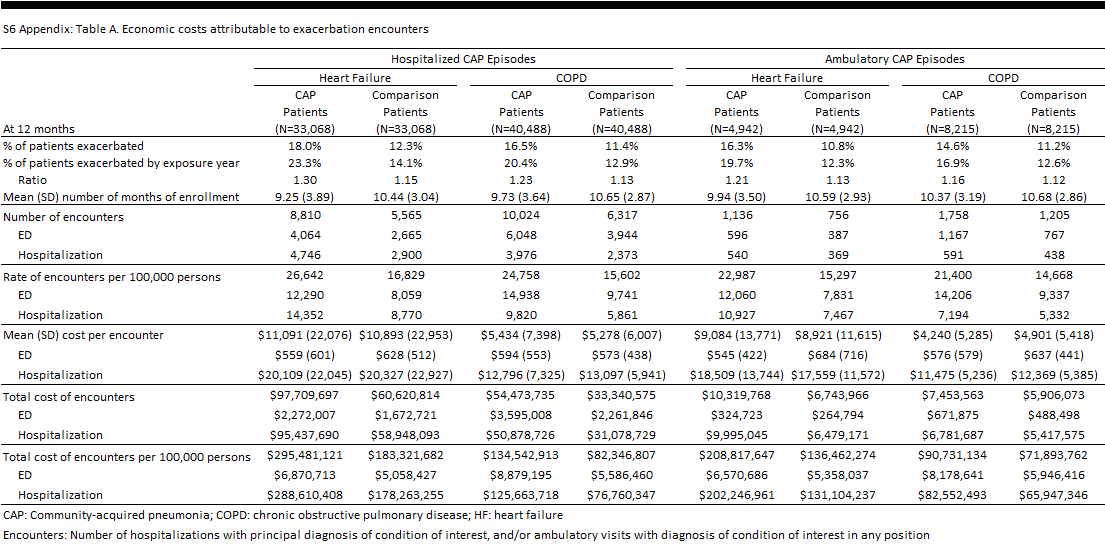

Supplement: S6 Appendix — Table A. Economic costs attributable to exacerbation encounters. (DOC) [file pone.0184877.s006.doc]
