# Supplementary material for: Risk of exacerbation following pneumonia in adults with heart failure or chronic obstructive pulmonary disease
Source: PLoS One. 2017 Oct 13;12(10):e0184877. doi: 10.1371/journal.pone.0184877 (PMC5640217; doi:10.1371/journal.pone.0184877)

ONLINE SUPPLEMENT 4 – APPENDIX:

**RESULTS FROM ANALYSES OF AMBULATORY CAP PATIENTS AND MATCHED COUNTERPARTS**


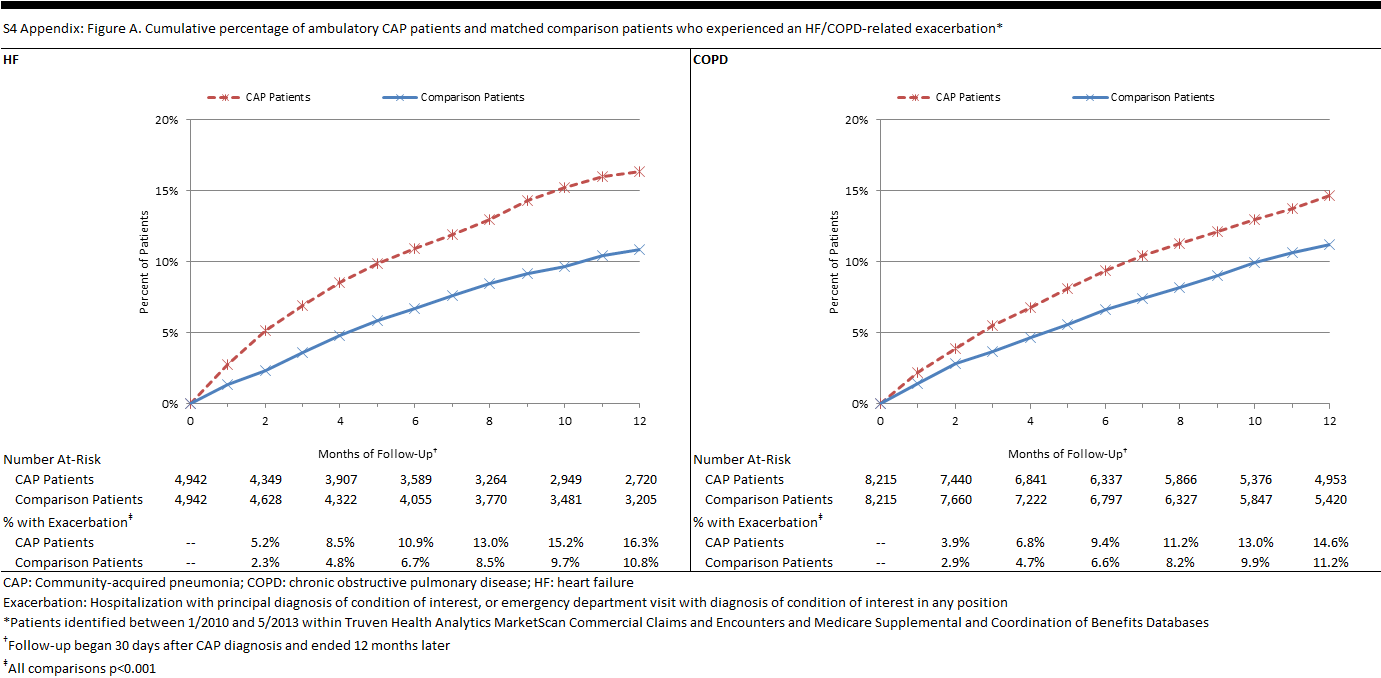


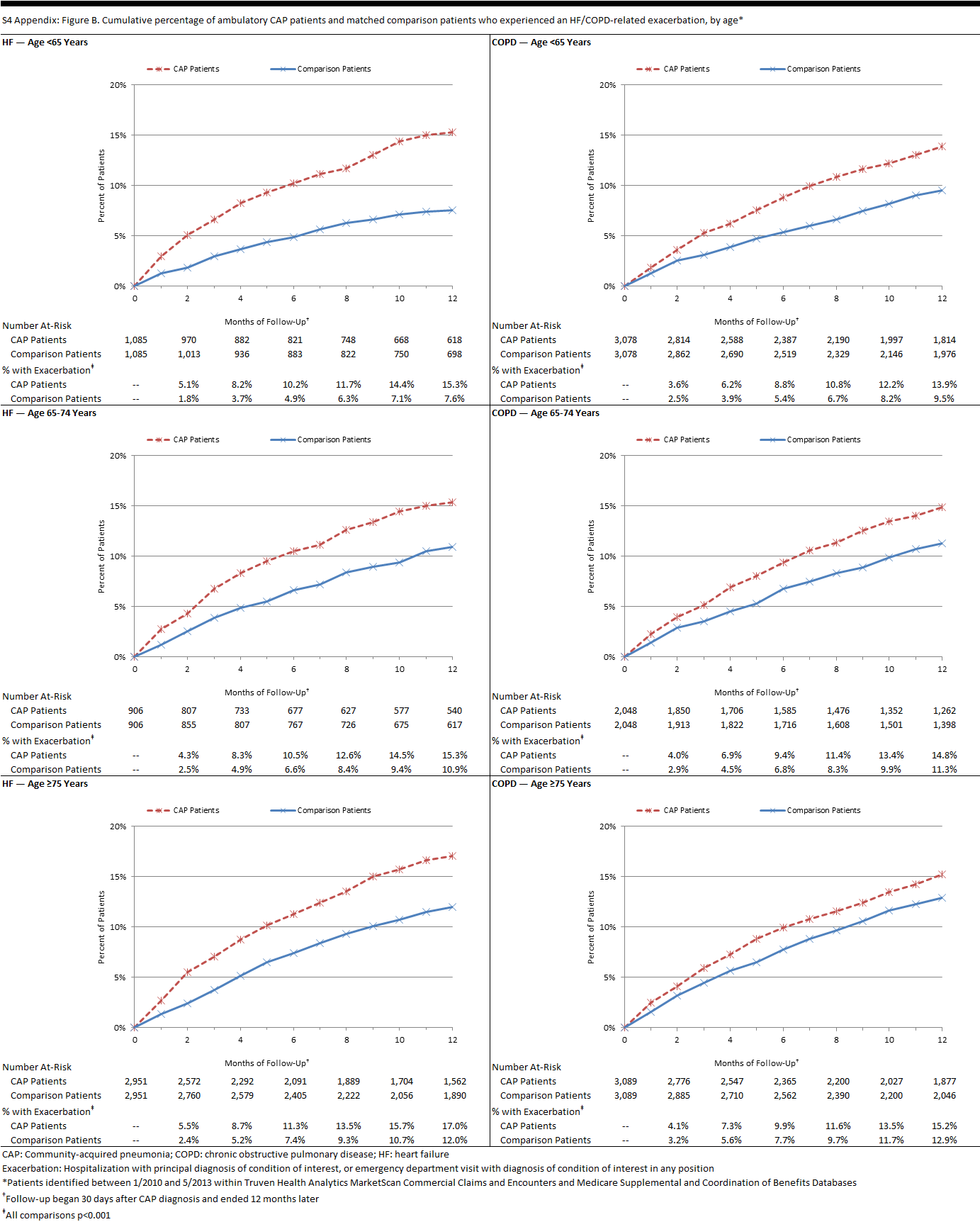


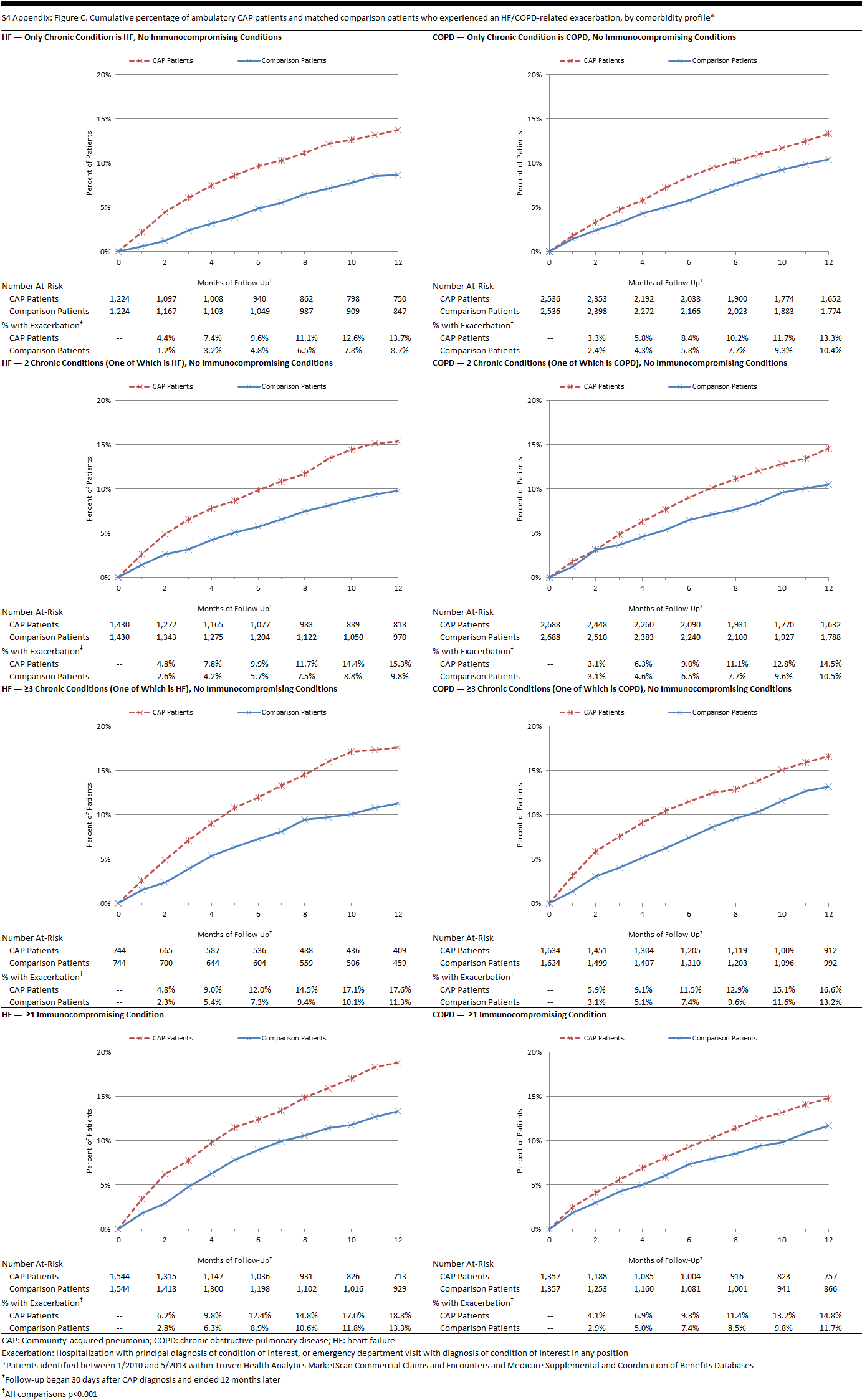

Supplement: S4 Appendix — Fig A. Cumulative percentage of ambulatory CAP patients and matched comparison patients who experienced an HF/COPD-related exacerbation, Fig B. Cumulative percentage of ambulatory CAP patients and matched comparison patients who experienced an HF/COPD-related exacerbation, by age, Fig C. Cumulative percentage of ambulatory CAP patients and matched comparison patients who experienced an HF/COPD-related exacerbation, by comorbidity profile. (DOC) [file pone.0184877.s004.doc]
